# Long-Term Antitumor CD8^+^ T Cell Immunity Induced by Endogenously Engineered Extracellular Vesicles

**DOI:** 10.3390/cancers13092263

**Published:** 2021-05-08

**Authors:** Flavia Ferrantelli, Francesco Manfredi, Chiara Chiozzini, Patrizia Leone, Andrea Giovannelli, Eleonora Olivetta, Maurizio Federico

**Affiliations:** 1 National Center for Global Health, Istituto Superiore di Sanità, Viale Regina Elena 299, 00161 Rome, Italy; flavia.ferrantelli@iss.it (F.F.); francesco.manfredi@iss.it (F.M.); chiara.chiozzini@iss.it (C.C.); patrizia.leone@iss.it (P.L.); eleonora.olivetta@iss.it (E.O.); 2 National Center for Animal Experimentation and Welfare, Istituto Superiore di Sanità, Viale Regina Elena 299, 00161 Rome, Italy; andrea.giovannelli@iss.it

**Keywords:** extracellular vesicles, antitumor vaccines, HPV16, CD8^+^ T cell immunity

## Abstract

**Simple Summary:**

The induction of an effective immune response against tumor cells is of a great benefit in the battle against cancers. We recently characterized a novel, safe, and cost-effective strategy to induce an efficient CD8^+^ T cell immune response against potentially whatever antigen. This technique is based on in vivo engineering of exosomes/extracellular vesicles (EVs), i.e., nanovesicles constitutively released by all healthy cells. Immunogenic EVs are generated by intramuscular injection of a DNA vector expressing an EV-anchoring protein fused with the antigen of interest. In this paper, we applied our vaccine platform to counteract the growth of tumors expressing antigens of Human Papilloma Virus (HPV). We demonstrated that this method is instrumental in curing mice already developing HPV-related tumors. In addition, cured mice were shown to resist a second tumor cell implantation. These results could be of relevance for a possible translation into the clinic of our technology.

**Abstract:**

We developed an innovative method to induce antigen-specific CD8^+^ T cytotoxic lymphocyte (CTL) immunity based on in vivo engineering of extracellular vesicles (EVs). This approach employs a DNA vector expressing a mutated HIV-1 Nef protein (Nef^mut^) deprived of the anti-cellular effects typical of the wild-type isoform, meanwhile showing an unusual efficiency of incorporation into EVs. This function persists even when foreign antigens are fused to its C-terminus. In this way, Nef^mut^ traffics large amounts of antigens fused to it into EVs spontaneously released by the recipient cells. We previously provided evidence that mice injected with a DNA vector expressing the Nef^mut^/HPV16-E7 fusion protein developed an E7-specific CTL immune response as detected 2 weeks after the second immunization. Here, we extended and optimized the anti-HPV16 CD8^+^ T cell immune response induced by the endogenously engineered EVs, and evaluated the therapeutic antitumor efficacy over time. We found that the co-injection of DNA vectors expressing Nef^mut^ fused with E6 and E7 generated a stronger anti-HPV16 immune response compared to that observed in mice injected with the single vectors. When HPV16-E6 and -E7 co-expressing tumor cells were implanted before immunization, all mice survived at day 44, whereas no mice injected with either void or Nef^mut^-expressing vectors survived until day 32 after tumor implantation. A substantial part of immunized mice (7 out of 12) cleared the tumor. When the cured mice were re-challenged with a second tumor cell implantation, none of them developed tumors. Both E6- and E7-specific CD8^+^ T immunities were still detectable at the end of the observation time. We concluded that the immunity elicited by engineered EVs, besides counteracting and curing already developed tumors, was strong enough to guarantee the resistance to additional tumor attacks. These results can be of relevance for the therapy of both metastatic and relapsing tumors.

## 1. Introduction

Eukaryotic cells spontaneously release vesicles of different sizes including apoptotic bodies (1–5 μm), microvesicles (50–1000 nm), and exosomes (50–200 nm) [1]. Microvesicles (also referred to as ectosomes) shed by plasma membrane, whereas exosomes are released after inward invagination of endosome membranes and the formation of intraluminal vesicles. Healthy cells constitutively release both exosomes and microvesicles, together referred to as extracellular vesicles (EVs). They are a relevant means of intercellular delivery of macromolecules, such as DNAs, RNAs, proteins, and lipids [2,3].

EVs are abundant, stable, and highly bioavailable to tissues. They find potential applications as diagnostic biomarkers, therapeutics, drug delivery vehicles, and functional cosmetics. Several EV-based anticancer immunotherapies have been under clinical trials for different indications [4,5]. Despite high expectations, clinical trials did not confirm the therapeutic efficacy of in vitro/ex vivo engineered EVs, mostly because of a number of drawbacks associated with functional reproducibility and loading of specific cargoes [6].

Cancer cells express a burden of new antigens as a result of their intrinsic genetic instability typical of malignant transformation and/or the expression of the etiologic cancer agents, as in the case of virus-induced malignancies. Transformed cells can produce antigens to which the host is tolerant (tumor-associated self-antigens), and/or antigens eliciting an immune response not effective enough to counteract the tumor development. The latter include the so-called “tumor specific neo-antigens” as well as antigens normally produced in immune-privileged tissues. Hence, establishing a method to induce an adaptive immune response against both tolerogenic and non-tolerogenic tumor-associated antigens (TAAs) and neo-antigens would be of great relevance for the design of novel antitumor therapeutic approaches.

Two types of cancer immunotherapy have emerged so far as being the most promising: (i) immune modulation through monoclonal antibodies referred to as immune checkpoint blockers (ICBs), and (ii) T cell-based cancer immunotherapy, including active vaccination and adoptive cell transfer [7].

Striking advances have been made in anticancer immunotherapy in the last decade due to the use of monoclonal antibodies referred to as ICBs [8,9], able to foster pre-existing immunity against both TAAs and neo-antigens. These successes definitely proved that cancer can be treated and cured by manipulating the immune system. However, the ICB-based strategy still suffers from some limitations, including intrinsic and/or acquired resistance, and development of hyper-immune activation. Immune dysregulation can associate with adverse events affecting several organs, including skin, gut, heart, lungs, and bone [10].

Cancer vaccines aim at inducing antitumor immunity through immune response against whole or part of tumor antigens. In this way, a de novo antitumor immunity can be established, and both potency and breadth of pre-existing immunity can be widened. These effects can be generated by synthetic peptides designed on the sequence of both allogenic and autologous tumor antigens, pre-conditioned autologous dendritic cells (DCs), and genetic vaccines (DNA/RNA/viral vectors) [11].

We developed a vaccine platform based on the high levels of uploading into (EVs) of a Human Immunodeficiency Virus-1 Nef mutant, referred to as Nef^mut^ [12]. In the Nef^mut^-based platform, the antigen of interest is expressed by a DNA vector as product of fusion to Nef^mut^. In the model we proposed, Nef^mut^ expressed by intramuscularly-injected DNA vehiculates the antigen into the EVs spontaneously released by muscle cells. The engineered EVs are free to circulate into the body and can reach compartments distal from the injection site [13,14]. When they enter professional antigen-presenting cells (APCs), the product of fusion is cross-presented, thereby inducing antigen-specific cytotoxic T lymphocytes (CTLs) [14,15,16,17,18,19]. No humoral response is produced, most likely a consequence of the highly efficient uploading into EVs of Nef^mut^-based fusion products which remain hidden and protected from external environment.

Data regarding both duration and efficacy against tumor challenge and re-challenge of a novel anti-HPV16 vaccine based on in vivo-engineered EVs are reported.

## 2. Materials and Methods

### 2.1. DNA Vector Synthesis

The pTargeT (Invitrogen, Thermo Fisher Scientific, Waltham, MA, USA) vectors expressing Nef^mut^, Nef^mut^/HPV16-E6 and Nef^mut^/HPV16-E7 were already described [14,19]. The HPV16-related sequences were optimized for the expression in eukaryotic cells, and the domains involved in the pathogenic interactions with cell protein targets were inactivated [19,20,21]. To express HPV16-E6 and -E7 alone, the respective ORFs were inserted in the Not I and Apa I sites of the pTargeT vector polylinker. Kozak sequences were inserted at the 5′ end, and a 6×His tag sequence (i.e., 5′ CACCATCACCATCACCAT 3′) was included at the 3′ end just before the stop codon. Sequences were synthesized by Eurofins Genomics, Konstanz, Germany.

### 2.2. Cell Cultures and Transfection

Both human embryonic kidney (HEK)293T (ATCC, CRL-11268) and TC-1 (a generous gift of prof. Wu, Johns Hopkins Medical Institutes, Baltimore, MD, USA) cells were grown in DMEM (Gibco, Thermo Fisher Scientific) plus 10% heat-inactivated fetal calf serum (FCS, Gibco). TC-1 cells are primary lung epithelial cells isolated from C57BL/6 mice, and immortalized through the transduction of a retroviral vector expressing both HPV16-E6 and -E7, following by the engineering with a vector expressing the activated human c-Ha-ras oncogene. Transfection assays were performed using Lipofectamine 2000 (Invitrogen, Thermo Fisher Scientific).

### 2.3. EV Isolation

Cells transfected with vectors expressing the Nef^mut^-based fusion proteins were washed 24 h later, and reseeded in medium supplemented with EV-deprived FCS. The supernatants were harvested from 48 to 72 h after transfection. EVs were recovered through differential centrifugations [22] by centrifuging supernatants at 500× *g* for 10 min, and then at 10,000× *g* for 30 min. Supernatants were harvested, filtered with 0.22 μm pore size filters, and ultracentrifuged at 70,000× *g* for l h. Pelleted vesicles were resuspended in 1× PBS, and ultracentrifuged again at 70,000× *g* for 1 h. Finally, pellets containing EVs were resuspended in 1:100 of the initial volume.

### 2.4. Western Blot Analysis

Western blot analyses of both cell lysates and EVs were carried out after resolving samples in 10% sodium dodecyl sulfate-polyacrylamide gel electrophoresis (SDS-PAGE). In brief, the analysis on cell lysates was performed by washing cells twice with 1× PBS (pH 7.4) and lysing them with 1× SDS-PAGE sample buffer. Samples were resolved by SDS-PAGE and transferred by electroblotting on a 0.45 μM pore size nitrocellulose membrane (**GE Healthcare Europe GmbH, Milan, Italy**) overnight using a Bio-Rad (Hercules, CA, USA) trans-blot device. For Western blot analysis of EVs, they were lysed and analyzed as described for cell lysates. For immunoassays, membranes were blocked with 5% non-fat dry milk in PBS containing 0.1% Triton X-100 for 1 h at room temperature, then incubated overnight at 4 °C with specific antibodies diluted in PBS containing 0.1% Triton X-100. Filters were revealed using 1:1000-diluted sheep anti-Nef antiserum ARP 444 (MHRC, London, UK), 1:500-diluted anti-β-actin AC-74 mAb from Sigma (St. Louis, MI, USA), 1:500 diluted anti-Alix H-270 polyclonal Abs from Santa Cruz (Dallas, TX, USA), and 1:1000 diluted 6AT18 anti-6×His-tag mAb (Sigma).

### 2.5. Mice Immunization

Six-week old C57 Bl/6 female mice were obtained from Charles River (Calco, Italy) and placed in the Central Animal Facility of the ISS, as approved by the Italian Ministry of Health, authorization n. 950/2018. DNA vector preparations were diluted in sterile 0.9% saline solution. Both quality and quantity of the DNA preparations were checked by 260/280 nm absorbance and electrophoresis assays. Each inoculum volume was injected into both quadriceps. For electroporation-related procedures, mice were anesthetized with isoflurane as prescribed in the Ministry authorization. Immediately after inoculation, electroporation was applied at the site of injection through the Agilepulse BTX (Holliston, MA, USA) device using a 4-needle array 4 mm gap, 5 mm needle length, with the following parameters: One pulse of 450 V for 50 µsec; 0.2 msec interval; one pulse of 450 V for 50 µsec; 50 msec interval; eight pulses of 110 V for 10 msec with 20 msec intervals. Immunizations were repeated identically 10 or 14 days later. For immunogenicity studies, fourteen days after the last immunization mice were sacrificed by cervical dislocation as recommended by the Ministry authorization protocol. Spleens were then explanted and placed into a 2 mL Eppendorf tubes filled with 1 mL of RPMI 1640 (Gibco), 50 µM 2-mercaptoethanol (Sigma). PBMCs were recovered from blood samples obtained by retro orbital bleeding. Red blood cells were eliminated through incubation with ACK lysing buffer (Gibco).

### 2.6. IFN-γ EliSpot Analysis

A total of 2.5 × 10^5^ live splenocytes were seeded in each microwell. Cultures were run in triplicate in EliSpot multiwell plates (Millipore, Burlington, MA, USA) pre-coated with the AN18 mAb against mouse IFN-γ (Mabtech, Nacka Strand, Sweden) in RPMI 1640 (Gibco) plus 10% FBS (Gibco) for 16 h in the presence or not of 5 µg/mL of the following peptides: E6 18–26: KLPQLCTEL [23]; 50−57: YDFAFRDL [23]; 109–117: RCINCQKPL [24]; 127–135: DKKQRFMNI [24], and E7: 49–57: RAHYNIVTF [23]; 67–75: LCVQSTHVD [24]. As a negative control, 5 µg/mL of the H2-K^b^-binding HCV-NS3 specific peptide ITQMYTNV [25] were used. More than 70% pure preparations of the peptides were obtained from UFPeptides, Ferrara, Italy, and JPT, Berlin, Germany. For cell activation control, cultures were treated with 10 ng/mL phorbol 12-myristate 13-acetate (PMA, Sigma) plus 500 ng/mL of ionomycin (Sigma). After 16 h, cultures were removed, and the wells incubated with 100 µL of 1 µg/mL of the R4-6A2 biotinylated anti-IFN-γ (Mabtech) for 2 h at room temperature (r.t.) Wells were then washed and treated for 1 h at r.t. with 1:1000 diluted streptavidine-ALP preparations from Mabtech. After washing, spots were developed by adding 100 µL/well of Sigma Fast BCIP/NBT. The spot-forming cells were finally analyzed and counted using an AELVIS EliSpot reader (Hannover, Germany).

### 2.7. Intracellular Cytokine Staining (ICS)

Splenocytes were seeded at 2 × 10^6^/mL in RPMI medium, 10% FCS, 50 µM 2-mercaptoethanol (Sigma), and 1 μg/mL brefeldin A (BD Biosciences, Franklin Lakes, NJ, USA). Control conditions were carried out either by adding 10 ng/mL PMA (Sigma) and 1 μg/mL ionomycin (Sigma), or with unrelated peptides. After 16 h, cultures were stained with 1 μL of LIVE/DEAD Fixable Aqua Dead Cell reagent (Invitrogen Thermo Fisher Scientific) in 1 mL of PBS for 30 min at 4 °C and washed twice with 500 μL of PBS. To minimize nonspecific staining, cells were pre-incubated with 0.5 μg of Fc blocking mAbs (i.e., anti-CD16/CD32 antibodies, Invitrogen/eBioscience, Thermo Fisher Scientific) in 100 μL of PBS with 2% FCS for 15 min at 4 °C. For the detection of cell surface markers, cells were stained with 2 μL of the following Abs: FITC conjugated anti-mouse CD3, APC-Cy7 conjugated anti-mouse CD8a, and PerCP conjugated anti-mouse CD4 (BD Biosciences, Franklin Lakes, NJ, USA) and incubated for 1 h at 4 °C. After washing, cells were permeabilized and fixed through the Cytofix/Cytoperm kit (BD Biosciences) as per the manufacturer’s recommendations. Thereafter, cells were stained for 1 h at 4 °C with 2 μL of the following Abs: PE-Cy7 conjugated anti-mouse IFN-γ (BD Biosciences), PE conjugated anti-mouse IL-2 (Invitrogen eBioscience), and BV421 anti-mouse TNF-α (BD Biosciences) in a total of 100 μL of 1× Perm/Wash Buffer (BD Biosciences). After two washes, cells were fixed in 200 μL of 1× PBS/formaldehyde (2% v/v). Samples were then acquired by a Gallios flow cytometer and analyzed using Kaluza software (Beckman Coulter, Brea, CA, USA).

Gating strategy was as follows: live cells as detected by Aqua LIVE/DEAD Dye vs. FSC-A, singlet cells from FSC-A vs. FSC-H (singlet 1) and SSC-A vs. SSC-W (singlet 2), CD3 positive cells from CD3 (FITC) vs. SSC-A, CD8 or CD4 positive cells from CD8 (APC-Cy7) vs. CD4 (PerCP). The CD8^+^ cell population was gated against APC-Cy7, PE, and BV421 to observe changes in IFN-γ, IL-2, and TNF-α production, respectively. Boolean gates were created in order to determine any cytokine co-expression pattern.

### 2.8. qRT-PCR

Total RNA was extracted from one million of both TC-1 and, as negative control, murine macrophage RAW 264.7 cells (ATCC, TIB71) with the TRIzol Reagent (Invitrogen, Thermo Fisher Scientific) following the manufacturer’s recommendations. One μg of total RNA was used to synthesize cDNA by employing the Reverse Transcription (RT) System kit (Promega, Madison, WI, USA). One aliquot (2 μL) of cDNA was then amplified using the oligonucleotide primers specific for HPV16-E6 gene (forward: 5′-AATGTTTCAGGACCCACAGG-3′, and reverse: 5′-TTGTTTGCAGCTCTGTGCAT-3′) and for the E7 gene (forward: 5′-CAAGTGTGACTCTACGCTTCGG-3′, and reverse: 5′-GTGGCCCATTAACAGGTCTTCCAA-3′). Genomic DNA contamination was checked by including conditions run in the absence of RT. The RT reaction was normalized by amplifying samples also for hypoxanthine guanine phosphoribosyltransferase (HPRT) as a house-keeping gene. The assay was performed by means of the SYBR Green RT-PCR kit (Qiagen, Hilden, Germany) and the Applied Biosystems 7500 Real-Time PCR System (Applied Biosystems, Foster City, CA, USA). The reaction mix for each sample comprised: 12.5 μL of SYBR Green mix, 8.5 μL of ultra-pure, tri-distilled water, 2 μL of cDNA, 1 μL of primer mix (20 nM of each primer). The PCR reactions were led at 95 °C for 15″, 60 °C for 30″, 72 °C for 1′, for 40 cycles. Data were collected during every elongation step (72 °C) and during final ramping (for specificity control), and analyzed by Applied Biosystems 7500 SDS software using the 2^−DDCt^ method.

### 2.9. Tumor Challenge and Re-Challenge

Six-week old C57 Bl/6 female mice were obtained from Charles River and placed in the Central Animal Facility of the ISS. TC-1 cells (a kind gift of Prof. Wu, Johns Hopkins University, Boston, MA, USA) were prepared at 2 × 10^6^ cells/mL in 1× PBS, and 100 µL were inoculated subcutaneously (s.c.). At the time of immunization, mice were anesthetized with isoflurane as prescribed by the Ministry authorization. Immunizations were carried out as here above described and repeated after 10 days. To isolate peripheral blood mononuclear cells (PBMCs), seven days after the second immunization 200 μL of blood were collected from each mouse through retro orbital bleeding. Tumor growth was monitored by visual inspection, palpation, and measurement of diameters by an electronic caliper, and volumes calculated as (length × width^2^)/2. Mice were sacrificed by cervical dislocation as recommended by the Ministry authorization protocol if in poor health or as soon as tumors reached the size of 1 cm^3^.

Nineteen weeks after the last immunization, six mice that had recovered from tumor growth were re-challenged with TC-1 cells under the same experimental modalities used for the first implantation. Briefly, tumor cells were prepared at 2 × 10^6^ cells/mL in 1×PBS, and s.c. inoculated into mice (100 µL). The cells were implanted s.c. in the opposite side respect to the previous cell implantation. As a control, two age-matched (i.e., seven-months old) naïve mice were injected with TC-1 cells with identical modalities.

Tumor growth was monitored as above described. Mice were sacrificed by cervical dislocation either as soon as the tumor reached the size of 1 cm^3^, or at the 19 weeks after tumor cell re-implantation.

### 2.10. Statistical Analysis

When appropriate, data are presented as mean ± standard deviation (SD). In some instances, data were analyzed by two-tailed Mann–Whitney U test, two-tailed unpaired Student’s T test, or log rank test. *p* < 0.05 was considered significant.

## 3. Results

### 3.1. Optimization of the CD8^+^ T Immunogenicity Induced by Nef^mut^-Based DNA Vectors through Electroporation

The intramuscular (i.m.) injection of DNA vectors expressing a plethora of both tumor and viral antigens fused to Nef^mut^ was shown to induce antigen-specific CD8^+^ T cell immune responses [14,15,16,17,18,19]. In the intent to optimize the immune response induced by engineered EVs, we evaluated the impact of electroporation (EP) procedures applied soon after DNA injections. As widely described, EP is expected to increase the intracellular DNA delivery [26]. Hence, with the Nef^mut^-based platform, EP would increase the amounts of engineered EVs released by recipient cells, and, by consequence, the overall CD8^+^ T cell immune response.

**Figure 1:**
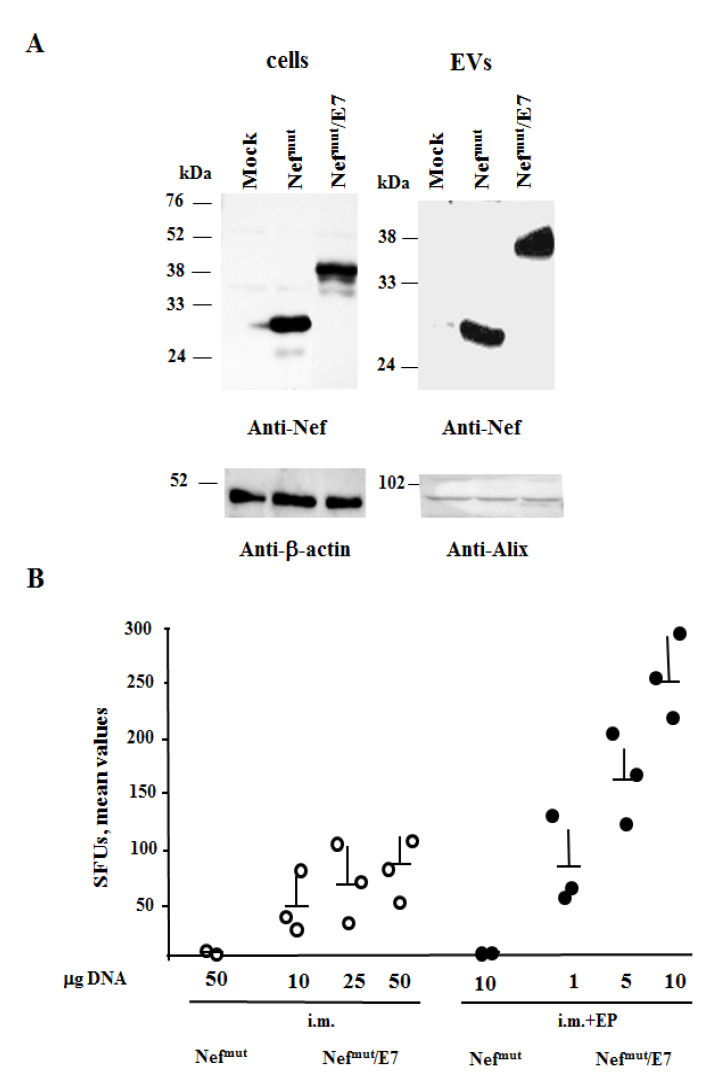
EP increases the antigen-specific CD8^+^ T cell response triggered by injection of Nef^mut^/E7 vector. (**A**) Detection of Nef^mut^/E7 in HEK293T transfected cells and EVs. Western blot analysis of 30 μg of lysates from cells transfected with DNA vectors expressing Nef^mut^/E7, and equal volumes of buffer where purified EVs were resuspended after differential centrifugations of the respective supernatants. As control, conditions from mock-transfected cells as well as cells transfected with Nef^mut^ were included. Polyclonal anti-Nef Abs served to detect Nef^mut^-based products, while β-actin and Alix were markers for cell lysates and EVs, respectively. Molecular markers are given in kDa. The results are representative of six independent experiments. (**B**) C57BL/6 mice (three per group) were injected i.m. with the indicated μg of DNA vectors expressing either Nef^mut^ or Nef^mut^/E7 followed or not by EP treatment. Treatments were repeated 14 days later, and after additional 14 days, splenocytes (2.5 × 10^5^/well) were tested in IFN-γ EliSpot assay for the presence of E7-specific CD8^+^ T lymphocytes. For each mouse, shown are the numbers of IFN-γ spot-forming units (SFUs) as mean values of triplicates after subtraction of values from wells treated with unrelated peptides.

Mice were injected i.m. in each quadricep with scaled doses of the DNA vector expressing either Nef^mut^ or Nef^mut^/E7 (whose EV uploading levels were previously tested upon cell transfection, Figure 1A and Figure S1), followed or not by local EP. Treatments were repeated 14 days later and, after additional 2 weeks, the anti-E7 response in splenic CD8^+^ T cells was measured. We observed that the DNA delivery by EP greatly increased E7-specific CD8^+^ T-cell responses (Figure 1B). Compared to i.m. injection with 50 μg of Nef^mut^/E7 expressing vector, Nef^mut^/E7 DNA combined with EP triggered an immune response about three-fold higher with a DNA dose five-fold lower.

We concluded that EP procedures increased the overall immunogenic effects induced by the injection of Nef^mut^-based DNA vectors.

### 3.2. Optimization of the HPV16-Specific CD8^+^ T Immunogenicity through Co-Injection of DNA Vectors Separately Expressing E6 and E7 Fused with Nef^mut^

Very recently, we provided evidence that the co-injection of up to three DNA vectors expressing diverse SARS-CoV-2 antigens fused with Nef^mut^ generated an additive CD8^+^ T cell immune response, in the absence of evident negative interference among the different immunogens [13]. We aimed at assessing whether a similar additive effect would take place with HPV16 antigens. To this aim, two DNA vectors expressing Nef^mut^/E6 and Nef^mut^/E7, respectively, were considered.

**Figure 2:**
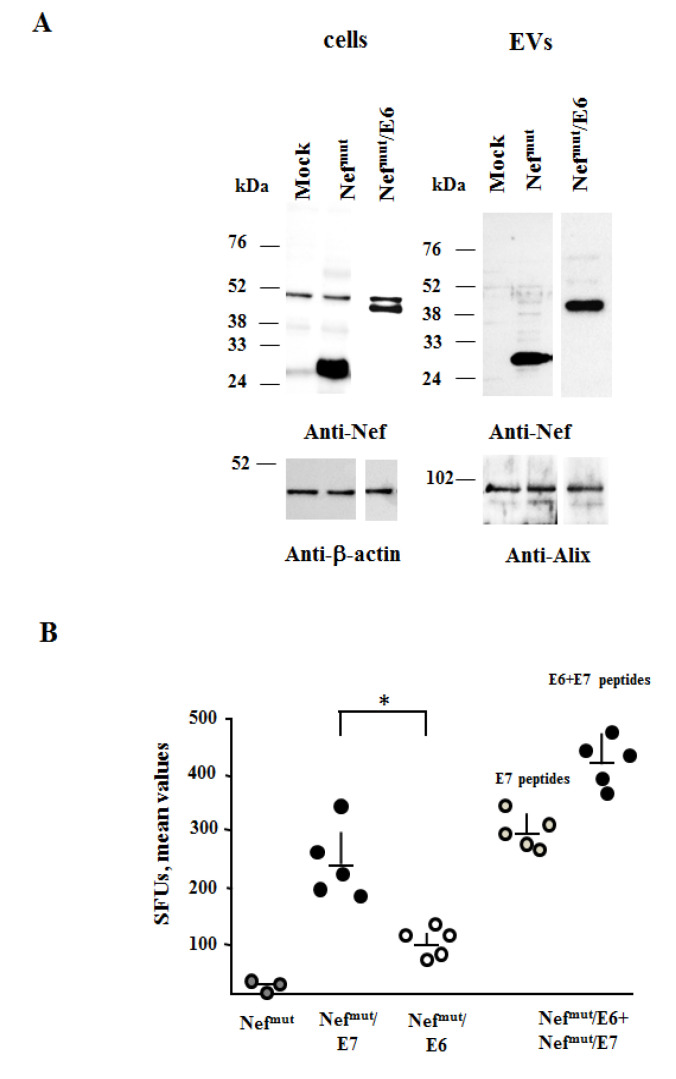
HPV16-E6 and -E7-specific CD8^+^ T cell immunity induced in mice co-injected with Nef^mut^/E6 and Nef^mut^/E7 DNA vectors. (**A**) Detection of Nef^mut^/E6 in HEK293T transfected cells and EVs. Western blot analysis of 30 μg of lysates from cells transfected with DNA vectors expressing Nef^mut^/E6, and equal volumes of buffer where purified EVs were resuspended after differential centrifugations of the respective supernatants. As control, conditions from mock-transfected cells as well as cells transfected with Nef^mut^ were included. Polyclonal anti-Nef Abs served to detect Nef^mut^-based products, while β-actin and Alix were markers for cell lysates and EVs, respectively. Molecular markers are given in kDa. The results are representative of three independent experiments. (**B**) CD8^+^ T cell immune response induced in C57 Bl/6 mice inoculated with the DNA vectors expressing Nef^mut^/E6 and Nef^mut^/E7 either singularly (10 μg) or in combination (10 μg each). As a control, mice were inoculated with 20 μg of the Nef^mut^-expressing vector. At the time of sacrifice, 2.5 × 10^5^ splenocytes were incubated o.n. with or without 5 μg/mL of either unrelated, E6, E7, or E6+E7-specific peptides in triplicate IFN-γ EliSpot microwells. For each mouse, shown are the numbers of IFN-γ SFUs as mean values of triplicates after subtraction of values from wells treated with unrelated peptides. The E7-specific immune response was evaluated also in co-injected mice. Intragroup mean values + SD are also reported. * indicates statistically significant differences.

By transfection in HEK293T cells, we assessed that both vectors expressed the fusion proteins, which were efficiently uploaded into EVs (Figure 1A, Figure 2A, Figures S1 and S2). DNA vectors were i.m. injected in mice either singularly or in combination. Fourteen days after the second immunization, the CD8^+^ T cell immune response was evaluated in terms of antigen-specific activation of CD8^+^ T cells through both IFN-γ EliSpot assay and ICS analysis.

Through the IFN-γ EliSpot assay, we observed that, when fused to Nef^mut^, E7 elicited stronger CD8^+^ T cell responses than E6 (Student’s T test, *p* = 0.0026), whereas the co-injection of both vectors generated an additive immune response (Figure 2B). Possible functional interferences can be excluded also by the evidence that in co-injected animals the E7-specific CD8^+^ T cell response was not lower than that detectable in mice injected with the Nef^mut^/E7-expressing vector alone (Figure 2B).

**Figure 3:**
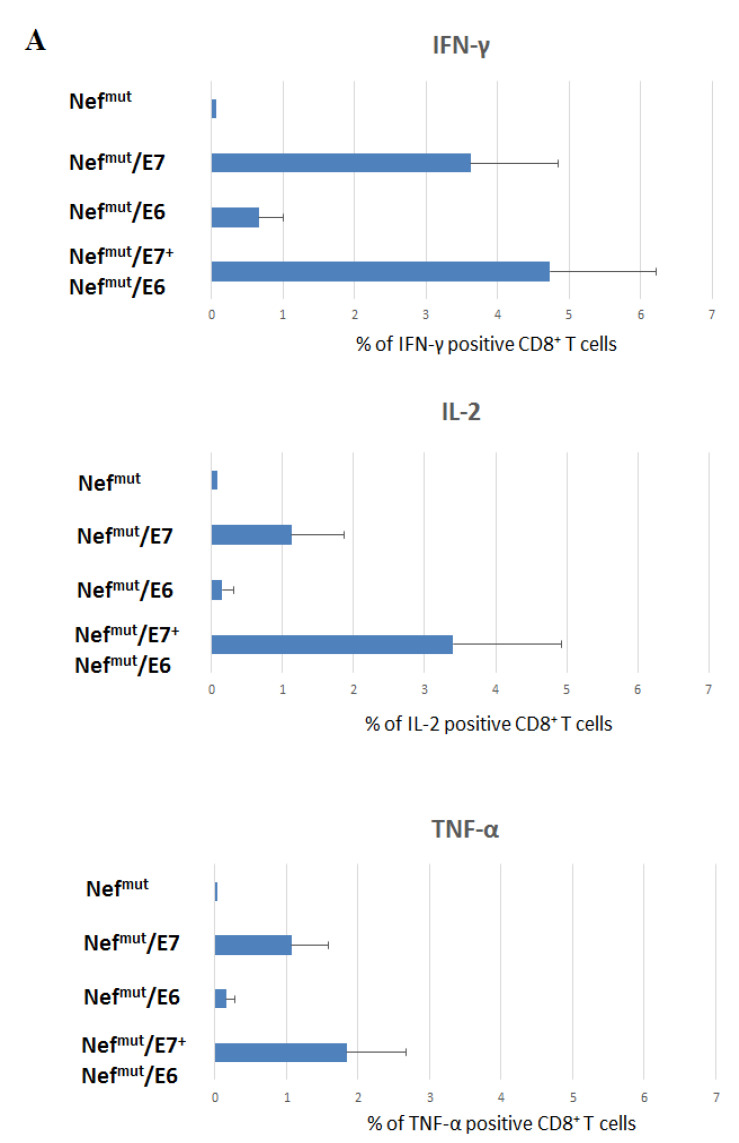

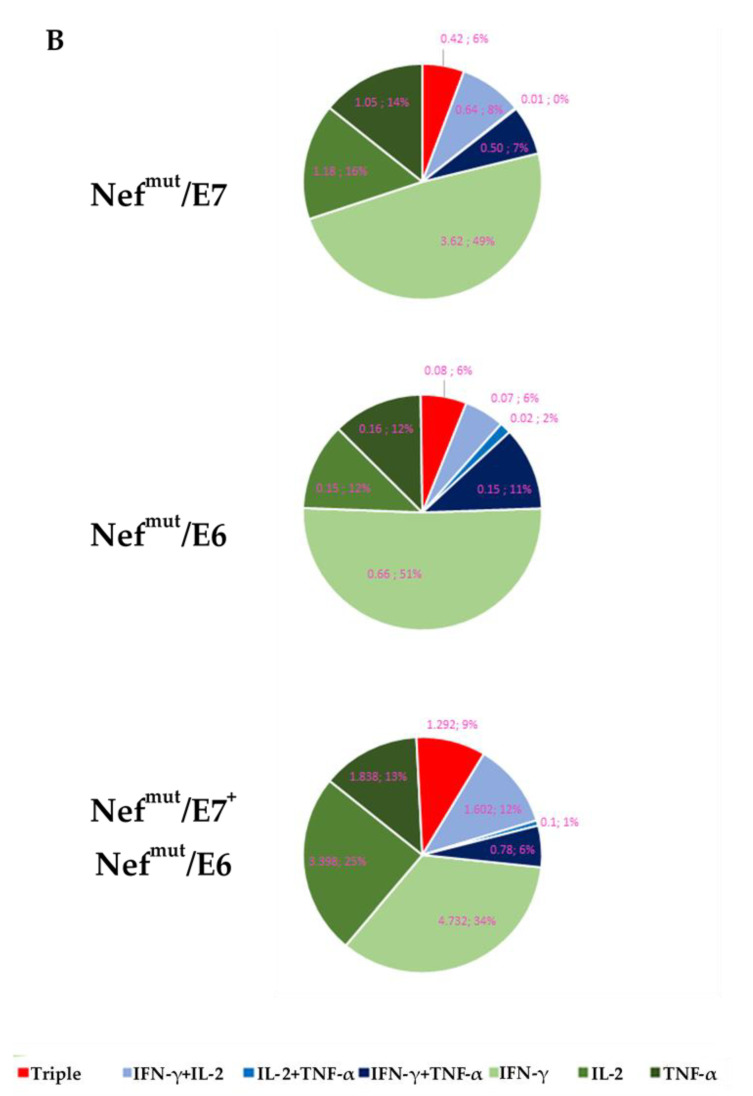
Intracellular cytokine staining analysis on CD8^+^ T cells from injected mice. (**A**) Percentages of CD8^+^ T cells expressing IFN-γ, IL-2, and TNF-α over the total of CD8^+^ T cells within splenocytes isolated from each mouse injected with the indicated DNA vectors. A total of 2.5 × 10^5^ splenocytes were incubated o.n. with or without 5 μg/mL of either unrelated or E6+E7-specific nonamers. Shown are mean values +SD of the absolute percentages of positive CD8^+^ T cells within total CD8^+^ T cells from cultures treated with specific peptides after subtraction of values detected in CD8^+^ T cells from cultures treated with unrelated peptide. (**B**) Pie charts indicating the means of both absolute (i.e., over the total of analyzed CD8^+^ T cells) and relative percentages of CD8^+^ T cells expressing cytokine combinations within pools of splenocytes from mice injected with the indicated vectors. Percentages were calculated after subtraction of values detected in homologous cultures treated with unrelated peptides. Values detected with splenocytes from mice injected with control vectors were below the sensitivity threshold of the assay. The results are representative of two independent experiments.

The generation of antigen-specific polyfunctional lymphocytes is a hallmark of efficacy for CD8^+^ T cell immune response. Therefore, we next evaluated the levels of E6- and/or E7-specific CD8^+^ T cells expressing IFN-γ, IL-2, and TNF-α by ICS after overnight cultivation of splenocytes with specific nonamers. Consistently with what observed by IFN-γ EliSpot assays, the immunization with the Nef^mut^/E7-expressing vector resulted in higher percentages of IFN-γ producing CD8^+^ T cells compared to those from Nef^mut^/E6 immunized mice (Figure 3A and Figures S3–S7). However, the highest percentages of IFN-γ producing CD8^+^ T cells were observed with splenocytes from mice injected with the two DNA vectors (Figure 3A). Similar results were obtained through the analysis of IL-2 and TNF-α expression. As expected based on the previous results, the percentages of triple-positive (i.e., polyfunctional) antigen-specific CD8^+^ T cells were higher in co-injected mice compared to mice injected with single vectors (Figure 3B).

We concluded that the optimal CD8^+^ T cell immune response can be achieved by co-injecting Nef^mut^/E6- and Nef^mut^/E7-expressing DNA vectors.

### 3.3. HPV16-E6 and –E7-Specific CD8^+^ T Cell Immune Response in Mice Immunized after Tumor Implantation

**Figure 4:**
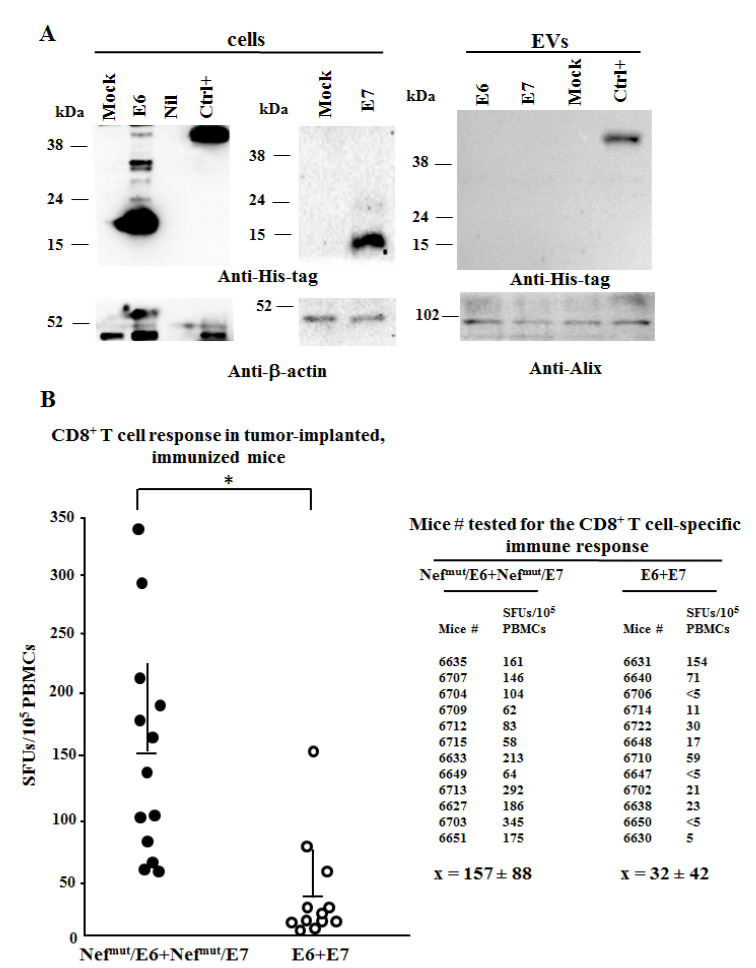
HPV16 E6- and E7-specific CD8^+^ T cell immunity induced in tumor-implanted mice. (**A**) Analysis of both HPV16-E6 and -E7 products in HEK293T transfected cells and respective EVs. Western blot analysis of 30 μg of lysates from cells transfected with DNA vectors expressing the indicated HPV16 ORFs (left panels), and equal volumes of buffer where purified EVs were resuspended after differential centrifugations of the respective supernatants (right panels). As control, conditions from mock-transfected cells as well as cells transfected with Nef^mut^ fused with a scFv (Nef^mut^/GO, Ctrl+) expressing a His-tag at its C-terminus were included. Polyclonal anti-His-tag Abs served to detect both HPV16-related and Nef^mut^/GO products, while β-actin and Alix were revealed as markers for cell lysates and EVs, respectively. Molecular markers are given in kDa. The results are representative of two independent experiments. (**B**) CD8^+^ T cell immune response in C57 Bl/6 mice implanted s.c. with 2 × 10^5^ TC-1 cells, and then inoculated+EP with 10 μg of DNA vectors expressing E6 and E7 either alone or fused with Nef^mut^. PBMCs were isolated after retro orbital bleeding, and then incubated o.n. with or without 5 μg/mL of either unrelated or E6+E7-specific nonamers in triplicate IFN-γ EliSpot microwells. Shown are the number of IFN-γ SFUs/10^5^ PBMCs as mean values of triplicates after subtraction of values from wells treated with unrelated peptides. On the right, the SFU values are associated with each mouse. Intragroup mean values + SD are also reported. * indicates statistically significant differences.

Next, the CD8^+^ T cell immune response against both E6 and E7 was tested in mice immunized after s.c. implantation of TC-1 tumor cells. The actual expression of both HPV16-E6 and -E7 genes in TC-1 cells was confirmed by qRT-PCR assay (Figure S8). C57 Bl/6 mice were implanted with 2 × 10^5^ TC-1 cells and, 10 days thereafter, mice bearing palpable tumors underwent the first immunization. Mice were injected with both Nef^mut^/E6- and Nef^mut^/E7-expressing vectors or, as control, with: (i) void DNA vector; (ii) a vector expressing Nef^mut^ alone, and (iii) a combination of vectors expressing E6 and E7. As expected, neither E6 nor E7 associated with EVs at detectable levels, as shown by the Western blot analysis of EVs isolated from the supernatants of cells transfected with either E6- or E7-expressing vectors. (Figure 4A and Figure S9). In this assay, a DNA vector expressing a His-tagged single-chain antibody (scFv) fused with Nef^mut^ [27] was used as control.

The immunizations were repeated after a week, and after additional seven days, 200 μL of blood were drawn to evaluate the immune responses. We found an about five-fold more potent immune response in mice injected with DNA vectors expressing the HPV16 proteins fused with Nef^mut^ than in those injected with vectors expressing E6 and E7 alone (Mann–Whitney U test, *p* = 0.0001) (Figure 4B). In this experimental setting, the implantation of the E6/E7-expressing TC-1 cells did not result per se in detectable CD8^+^ T cell immune responses.

We concluded that the levels of immune responses against the antigens fused to Nef^mut^ were in line with previous observations made in tumor-free mice.

### 3.4. Antitumor Therapeutic Effect in Mice Co-Injected with Vectors Expressing E6- and E7-Based Fusion Proteins

**Figure 5:**
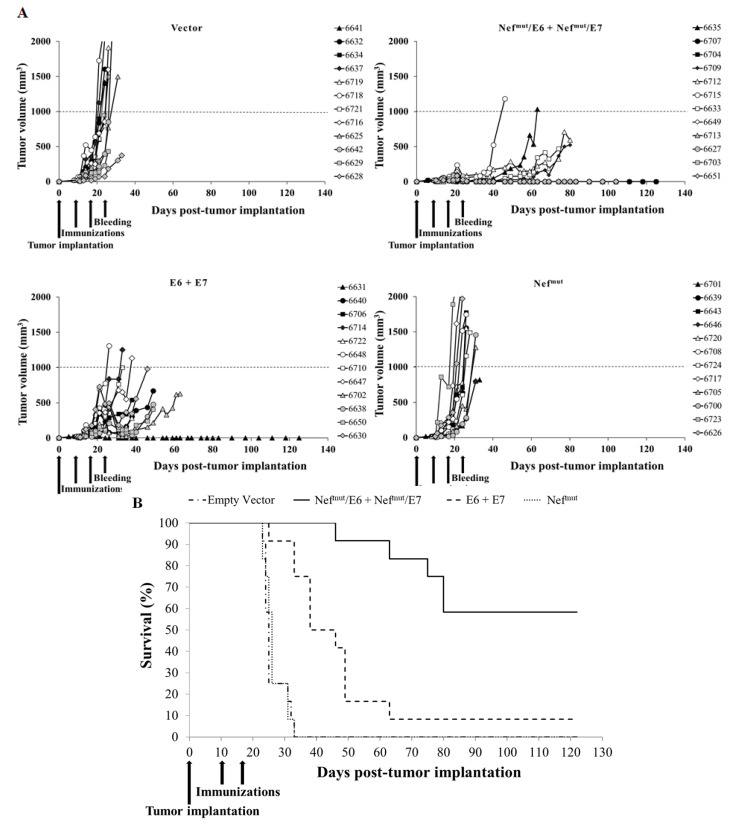
Antitumor therapeutic effect induced by i.m. injection of both Nef^mut^/E6 and Nef^mut^/E7 DNA vectors. (**A**) Tumor growth curves. C57 Bl/6 mice (12 per group) were challenged with 2 × 10^5^ TC-1 cells and, in the presence of palpable tumor masses, co-inoculated with the indicated DNA vectors. The DNA inoculations were repeated at day 17 after tumor cell implantation, and the growth of tumor mass was followed over time. Shown are data referred to each injected mouse identified by the different symbols. Tumor sizes were measured every 2–3 days. X-axis scale indicates the days of tumor monitoring, as well as the timing of tumor implantation, immunization and bleeding. (**B**) Kaplan–Meier survival curve.

Next, to evaluate the efficacy of the CD8^+^ T cell immune responses, the tumor size in injected mice was evaluated over time. Tumors implanted in mice injected with either void or Nef^mut-^expressing vectors grew in a very quick and uncontrolled way, killing the mice rapidly (Figure 5A). Tumors developed more slowly in mice injected with both vectors expressing E6 and E7. In this group, the mouse occasionally showing a strong CD8^+^ T cell response remained tumor-free, while in remainders the tumors reached the maximum size allowed before euthanasia (i.e., 1 cm^3^) within 60 days (Figure 5A). Conversely, seven of the 12 mice immunized with both Nef^mut^/E6 and Nef^mut^/E7-expressing vectors were cured and remained tumor-free. In this group, at day 35 after tumor implantation (i.e., when all mice of control groups had been euthanized) no or very limited tumor growth was observed. At day 65 after tumor implantation, when only a single mouse injected with vectors expressing E6 and E7 remained alive, all mice immunized with Nef^mut^/E6 plus Nef^mut^/E7 still survived (Figure 5B), and the differences between Kaplan–-Meier survival curves relative to these two groups of mice were statistically significant (log-rank test, *p* = 0.0005). In the Nef^mut^/E6 plus Nef^mut^/E7 group, a good association between the levels of antigen-specific CD8^+^ T cell immune responses as detected seven days after the second immunization and the antitumor effect was found. In fact, the mean of SFUs/10^5^ PBMCs measured in mice developing tumor was 115 ± 61, whereas in cured mice it reached 187 ± 92.

Taken together, these data demonstrated that the Nef^mut^/E6 plus Nef^mut^/E7 combined vaccine led to a tumor growth control much more potent than that induced by the combined injection of DNA vectors expressing HPV16-E6 and -E7.

### 3.5. Cured Mice Resisted the Tumor Re-Challenging

**Figure 6:**
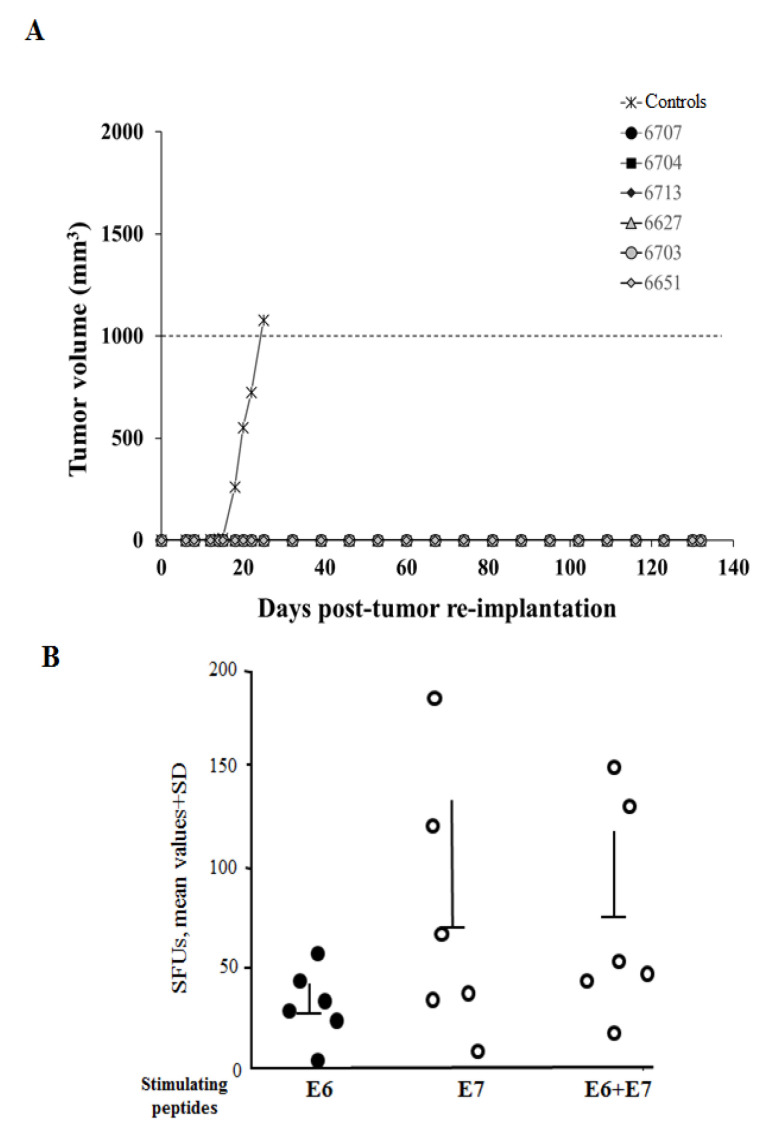
Antitumor therapeutic effect against re-implanted TC-1 cells. (**A**) Tumor growth curves. Cured C57 Bl/6 mice were re-challenged with 2 × 10^5^ TC-1 cells, and the growth of tumor masses were followed over time. As control, age-matched naïve mice were inoculated with the same number of cells, and the mean of tumor volumes are reported. Tumor sizes were measured every 2–3 days. The identification number of each injected mouse is shown. (**B**) CD8^+^ T cell immunity in mice challenged, immunized, and re-challenged with TC-1 cells. Nineteen weeks after cell re-implantation, mice were sacrificed, and splenocytes tested for E6- and/or E7-specific CD8^+^ T cell immunity by IFN-γ EliSpot assay. The number of IFN-γ SFUs/well are shown as mean values of triplicates after subtraction of values from wells treated with unrelated peptides. Intragroup mean values + SD are reported.

Next, we were interested in evaluating the persistence of the antitumor state in mice cured by the immunization with DNA vectors expressing Nef^mut^/E6 and Nef^mut^/E7. Six cured mice were re-challenged by injecting 2 × 10^5^ TC-1 cells in the opposite flank respect to the previous cell implantation. As control, age-matched, non-immunized mice were used. We observed that cured mice remained tumor-free over the 135 days of monitoring, whereas the age-matched naïve control mice developed a palpable tumor after 12 days from cell implantation, and had to be sacrificed at day 20 (Figure 6A). The six tumor-free animals were sacrificed at the end of follow-up, and their splenocytes tested by IFN-γ Elispot assay to assess the presence of E6- and/or E7-specific CD8^+^ T cells. Detectable levels of both E6- and E7-specific CD8^+^ T cell immune responses were still observed, with a prevalent E7-specific immunity (Figure 6B). All re-challenged mice were confirmed tumor-free and with normal-looking organs (i.e., lungs, heart, liver, spleen, kidneys, stomach, and gut) by observation at necroscopy.

These results indicated that the antitumor state induced by engineered EVs was strong and durable enough to counteract the proliferation of re-challenging tumor cells.

## 4. Discussion

Duration and robustness of the immune response are key features for any vaccine. We previously demonstrated the induction of strong CD8^+^ T cell immunity towards a wide range of both tumor and viral antigens through the Nef^mut^-based vaccine platform [14,15,16,17,18,19]. However, the long-term efficacy against tumor cell challenge and re-challenge had not been evaluated yet. To fill this gap, the transplantable tumor model based on TC-1 cells was considered. We first established the protocol to generate the most efficient immune response against both HPV16 E6 and E7. We found that associating EP with co-injection of DNA vectors expressing the two antigens fused to Nef^mut^ resulted in the most efficient immune response and production of the highest percentages of antigen-specific polyfunctional CD8^+^ T lymphocytes.

In tumor-implanted mice, the co-injection of Nef^mut^ –based vectors elicited a CD8^+^ T cell immune response much higher than that elicited in mice injected with vectors expressing the HPV16 products alone and, consistently, the antitumor effect was far more effective. Results from the tumor re-challenge experiment demonstrated that the Nef^mut^/E6 plus Nef^mut^/E7 vaccine conferred a potent, long lasting (at least up to 38 weeks after the last immunization), and effective CD8^+^ T cell immunity, which acted against the tumors re-implanted 19 weeks after the last immunization. All mice remained tumor-free, and the immune responses against both HPV16-E6 and -E7 proteins remained detectable at the end of the observation time.

Taken together, these data prove that the vaccine strategy based on endogenously engineered EVs is a promising approach to cure HPV-expressing tumors, and that it may be worth further testing against additional pathologies.

We believe that the here presented data represent an obvious advancement compared to the previously described therapeutic effect detected in mice injected with a Nef^mut^/E7 DNA vector [14]. In the previous experimental setting, the tumor monitoring was limited to 30 days after tumor implantation, and the in vivo tumorigenicity of the implanted TC-1 cell strain appeared significantly reduced compared to that observed in the here presented experiments. In fact, in previous experiments all mice injected with control vectors survived at day 30 after tumor implantation, with a tumor mass not exceeding 0.6 cm^3^. Conversely, in the here-presented experiment all mice injected with control vectors had to be euthanized between day 23 and 33 due to the overwhelming tumor growth. Most likely, this discrepancy depended on the implantation of cells with a high number of in vitro passages (i.e., more than 20). Conversely, here presented data were obtained with TC-1 cells just explanted from a naïve C57 Bl/6 mouse developing sub cute tumor.

Here reported results refer to a therapeutic antitumor design. The less effective vaccination based on in vitro engineered Nef^mut^/E7 EVs showed prophylactic [17] but not therapeutic antitumor effects (unpublished results). On this basis, it is conceivable that the here described vaccination strategy would have an antitumor prophylactic effect as well.

In the most accepted mechanism of action following i.m. DNA vaccination, antigens expressed by recipient muscle cells can be either exposed on cell membrane or secreted, thereby generating a humoral adaptive immune response [28]. Despite the many potential advantages offered by DNA vaccination, however, to date the efficacy in clinical trials has been disappointing, and it is uncertain whether the high expectations will be fulfilled [29]. In any case, preclinical and clinical studies have yielded many safety data [30].

To optimize the efficiency of engineered EV generation, in vivo delivery of vectors encoding the fusion constructs were enhanced via EP following i.m. DNA injection. In EP, the application of short pulsed electric fields increases the permeability of the cell plasma membrane to nucleic acids over naked DNA delivery alone through mechanisms not completely understood [31]. Furthermore, an increasing number of studies have shown that EP creates a local inflammatory environment that promotes the recruitment of immune cells, including macrophage and dendritic cells [32]. These additional immunological effects of EP are key aspects for immunity in response to vaccines. Currently, there are 242 clinical trials listed at clinicaltrials.gov with EP as part of the protocol.

Differently from the mechanisms associated with already-developed DNA vaccines, the vector-expressed Nef^mut^-derivatives are incorporated into the EVs spontaneously released by muscle cells, and are expected to freely circulate into the body. In this way, immunogenic EVs have the potential to be captured in distal lymph nodes, thereby eliciting the immunity also in compartments poorly replenished by circulating lymphocytes, as recently proven by analyzing the immunity in respiratory airways [13,19].

The Nef^mut^-based vaccine platform combines the remarkable benefits of efficient cross-presentation of EV-associated antigens and the consequent induction of a potent CD8^+^ T immune response, with several advantages typical of DNA vaccines, including: (i) simple and flexible design, so that a wide range of antigens and immunomodulatory molecules can be expressed; (ii) unrestricted MHC Class I immune response; (iii) no unsafe infectious agents involved in the vector preparation; (iv) great heat stability and ease of storage/transport without need for a cold chain; and (v) cost effectiveness, being the production of Nef^mut^-based vectors very rapid, reproducible, and perfectly suitable for large-scale administration.

Patient-derived EVs were employed as an innovative cancer immunotherapy in several clinical trials, but this strategy did not reach sufficient efficacy [33,34,35]. Other lines of research have focused on modifying the content and function of EVs in various ways, toward the end-goal of specialized therapeutic EVs [36]. Despite high expectations, however, clinical trials have not yet confirmed therapeutic applicability of ex vivo/in vitro engineered EVs [37,38,39]. Additionally, considering the underlying mechanism of action, the Nef^mut-^based approach can overcome the limitations pertaining DNA vaccines as well as vaccines based on ex vivo/in vitro engineered EVs.

## 5. Conclusions

The CD8^+^ T cell immunity generated by injection of Nef^mut^-based DNA vectors can cure already established tumors, and is both strong and lasting enough to induce resistance to additional tumor challenges. Effectiveness, simplicity, flexibility, and safety are the qualities rendering the Nef^mut^-based vaccine platform candidate for clinical applications.

## Data Availability

The data presented in this study are available on request from the corresponding author. The data are not publicly available due to patent application.

## Supplementary Materials

The following are available online at https://www.mdpi.com/article/10.3390/cancers13092263/s1, Figure S1: Western blot analysis for the expression of Nef^mut^/E7 in HEK293T cells and respective EVs. Raw data, Figure S2: Western blot analysis for the expression of Nef^mut^/E6 in HEK293T cells and respective EVs. Raw data, Figure S3: Gating strategy carried out in flow cytometry analysis of splenocytes from injected mice, Figure S4: Representative flow cytometer plots from the analysis of splenocytes isolated from Nef^mut^ injected mice, and treated with either MHC Class I-matched, unrelated nonamers or HPV16 E6/E7-specific nonamers, Figure S5: Representative flow cytometer plots from the analysis of splenocytes isolated from Nef^mut^/E6 injected mice, and treated with either MHC Class I-matched, unrelated nonamers or HPV16 E6-specific nonamers, Figure S6: Representative flow cytometer plots from the analysis of splenocytes isolated from Nef^mut^/E7 injected mice, and treated with either MHC Class I-matched, unrelated nonamers or HPV16 E7-specific nonamers, Figure S7: Representative flow cytometer plots from the analysis of splenocytes isolated from mice injected with Nef^mut^/E6 and Nef^mut^/E7 vectors, and treated with either MHC Class I-matched, unrelated nonamers or HPV16 E6+E7-specific nonamer, Figure S8: HPV16-E6 and -E7 gene expression in TC-1 cells, Figure S9: Western blot analysis for the expression of HPV16-E6 and -E7 in HEK293T cells and respective EVs. Raw data.
