# Supplementary material for: Long-Term Antitumor CD8+ T Cell Immunity Induced by Endogenously Engineered Extracellular Vesicles
Source: Cancers (Basel). 2021 May 8;13(9):2263. doi: 10.3390/cancers13092263 (PMC8125873; doi:10.3390/cancers13092263)
Supplement: Supplementary file 1 [file cancers-13-02263-s001.zip › cancers-1188757-supplementary.pdf]

cancers

MDPI

Supplementary Material: Long-Term Antitumor CD8+ T Cell Immunity Induced by Endogenously Engineered Extracellular Vesicles

Flavia Ferrantelli, Francesco Manfredi, Chiara Chiozzini, Patrizia Leone, Andrea Giovannelli, Eleonora Olivetta and Maurizio Federico

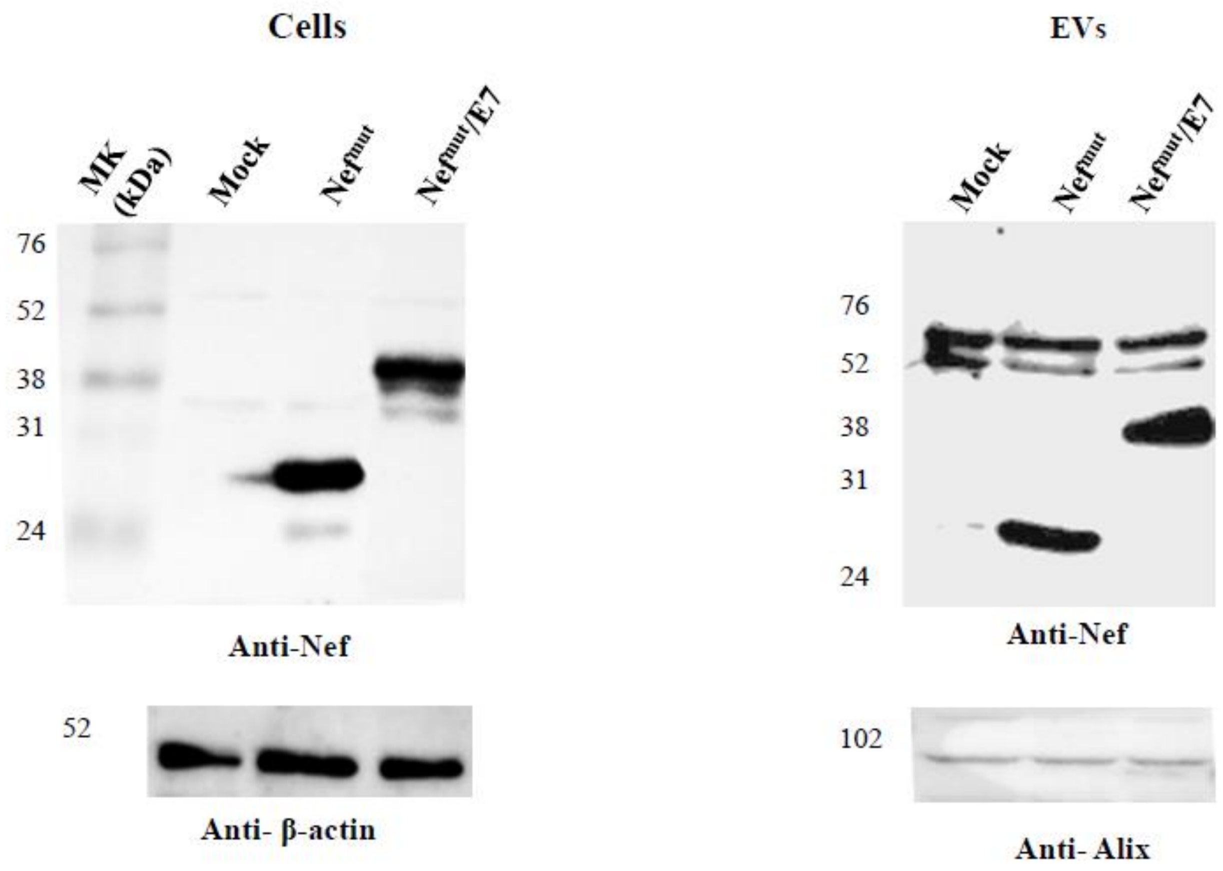

Figure S1. Western blot analysis for the expression of Nefmut/E7 in HEK293T cells and respective EVs. Raw data.

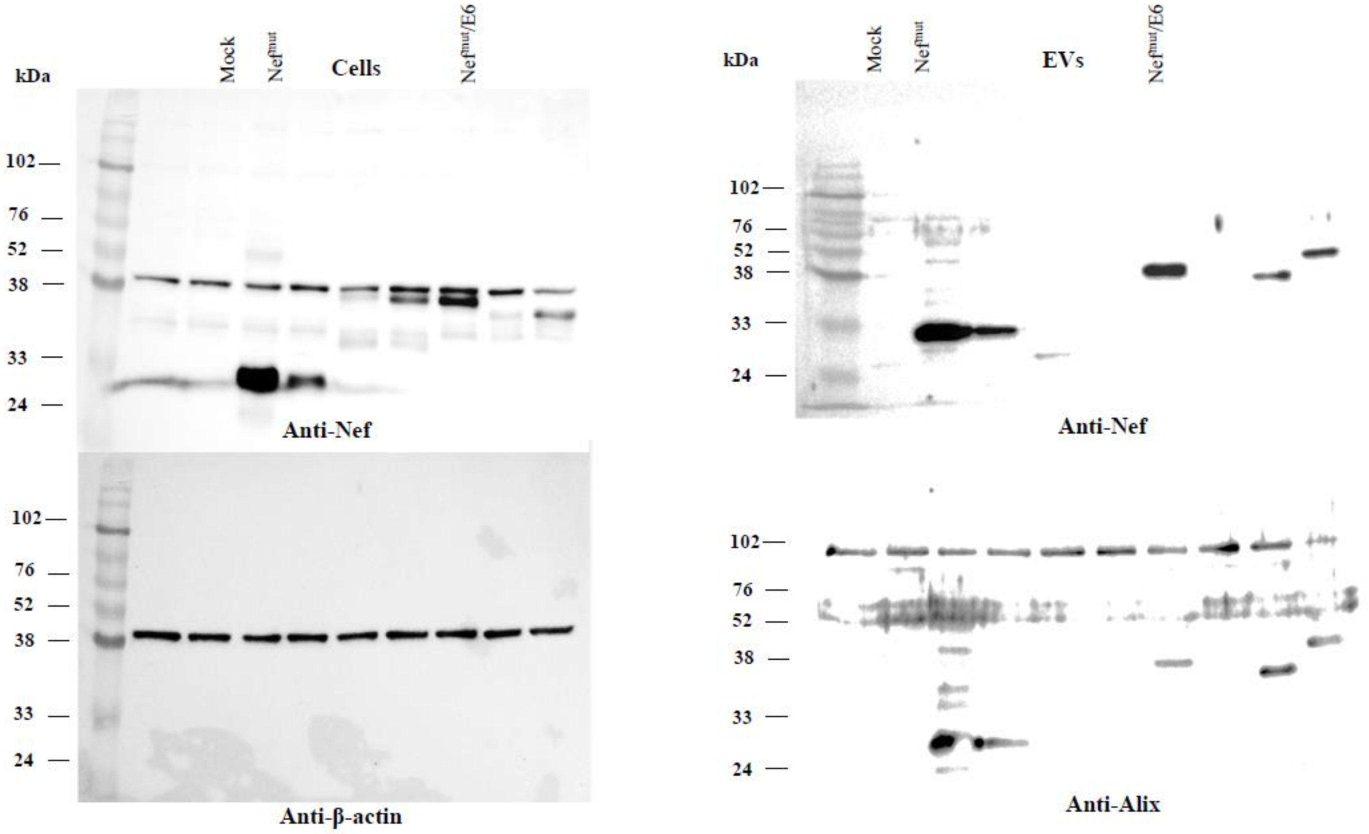

Figure S2. Western blot analysis for the expression of Nefmut/E6 in HEK293T cells and respective EVs. Raw data.

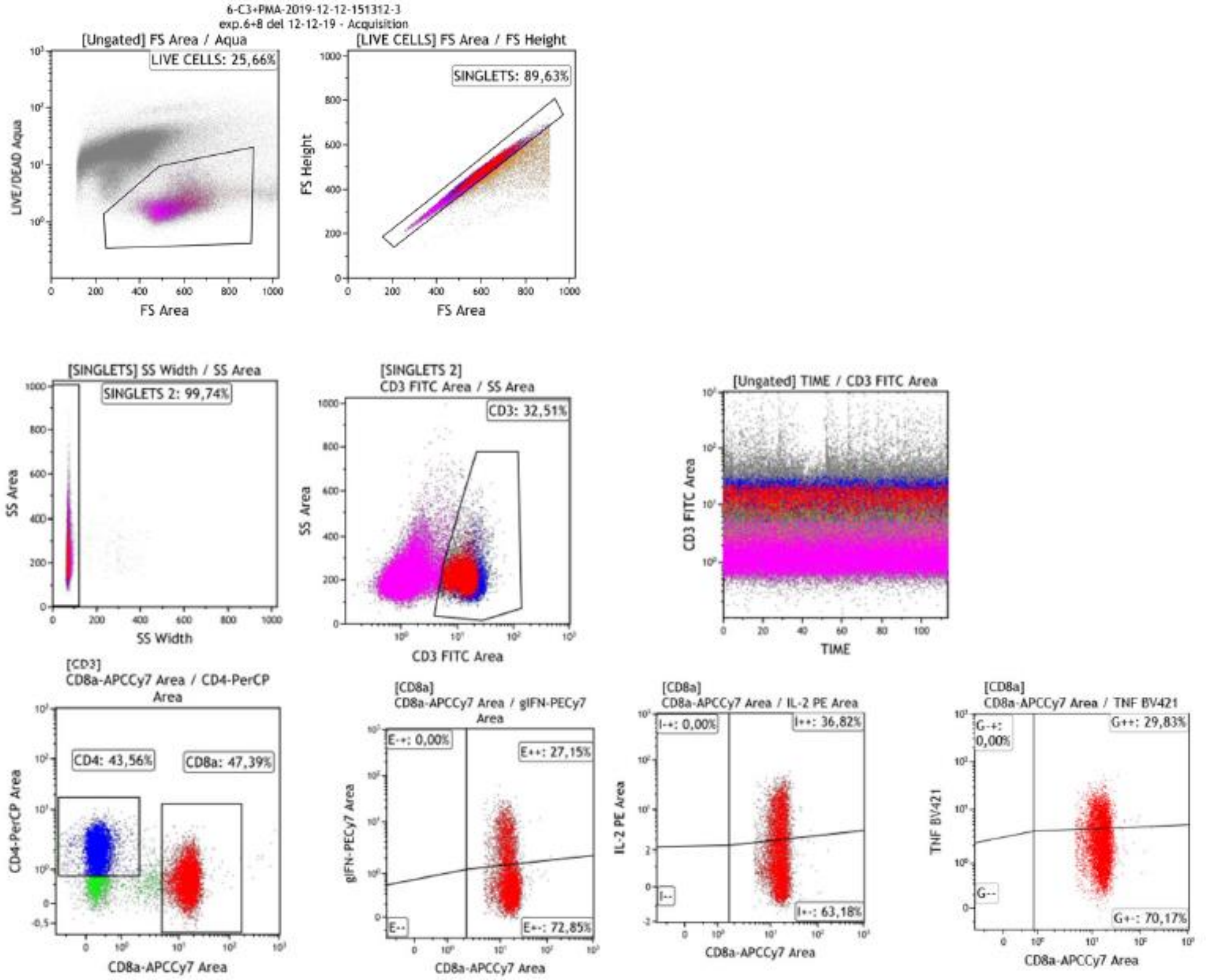

Figure S3. Gating strategy carried out in flow cytometry analysis of splenocytes from injected mice. shown is the analysis on PMA-treated cells.

Splenocytes from Nef^{mut} injected mice

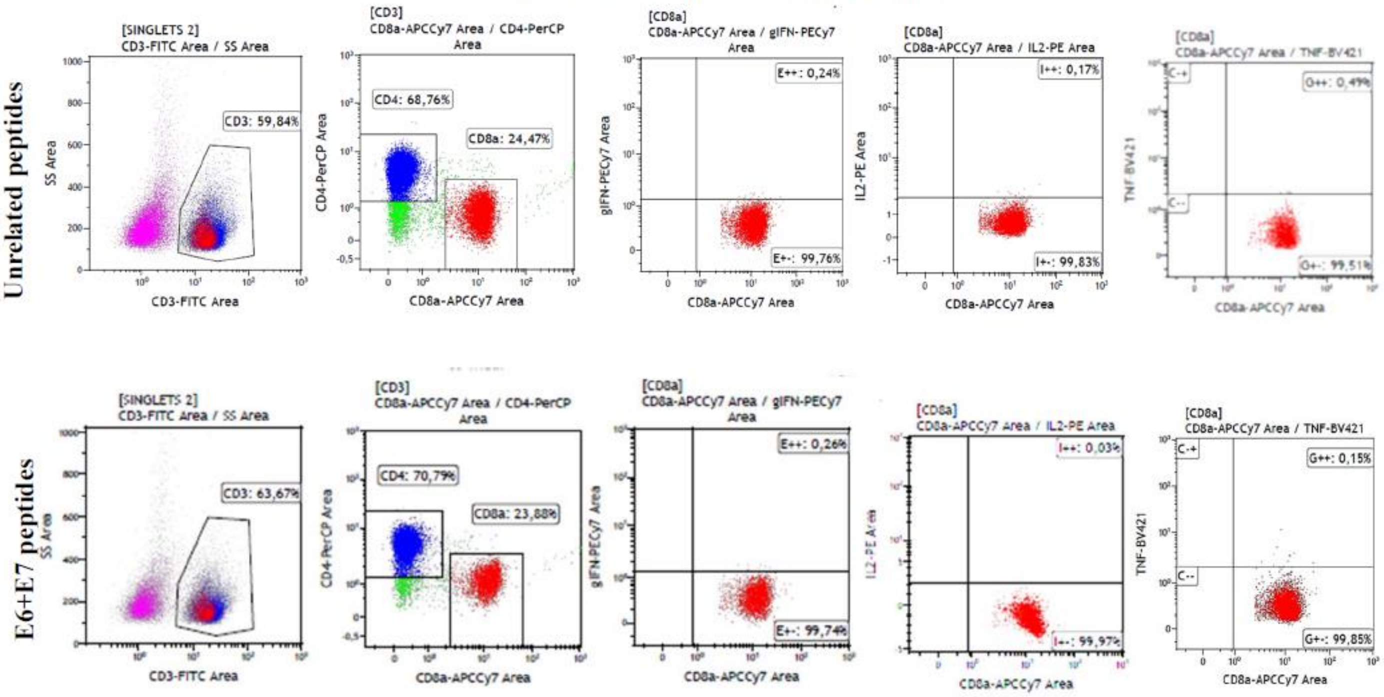

Figure S4. Representative flow cytometer plots from the analysis of splenocytes isolated from Nefmut injected mice, and treated with either MHC Class I-matched, unrelated nonamers (upper plots) or HPV16 E6/E7-specific nonamers (lower plots).

Splenocytes from Nef^{mut}/E6 injected mice

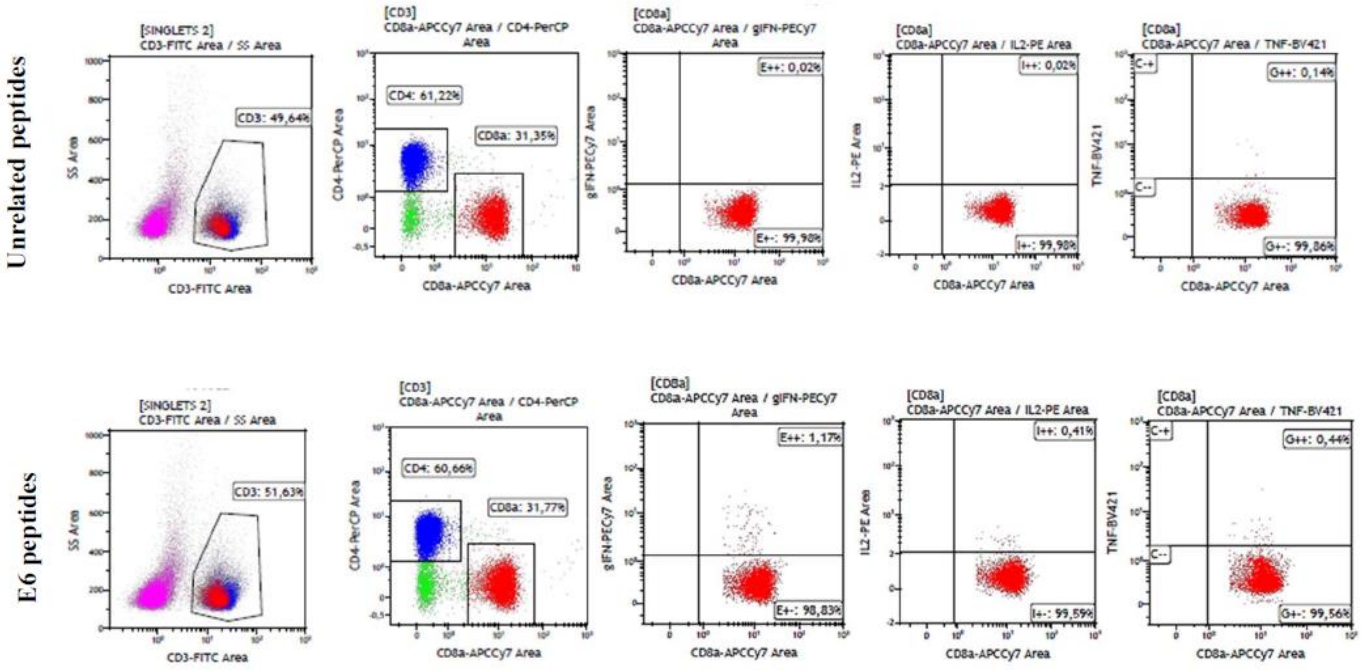

Figure S5. Representative flow cytometer plots from the analysis of splenocytes isolated from Nefmut/E6 injected mice, and treated with either MHC Class I-matched, unrelated nonamers (upper plots) or HPV16 E6-specific nonamers (lower plots).

Splenocytes from Nef^{mut}/E7 injected mice

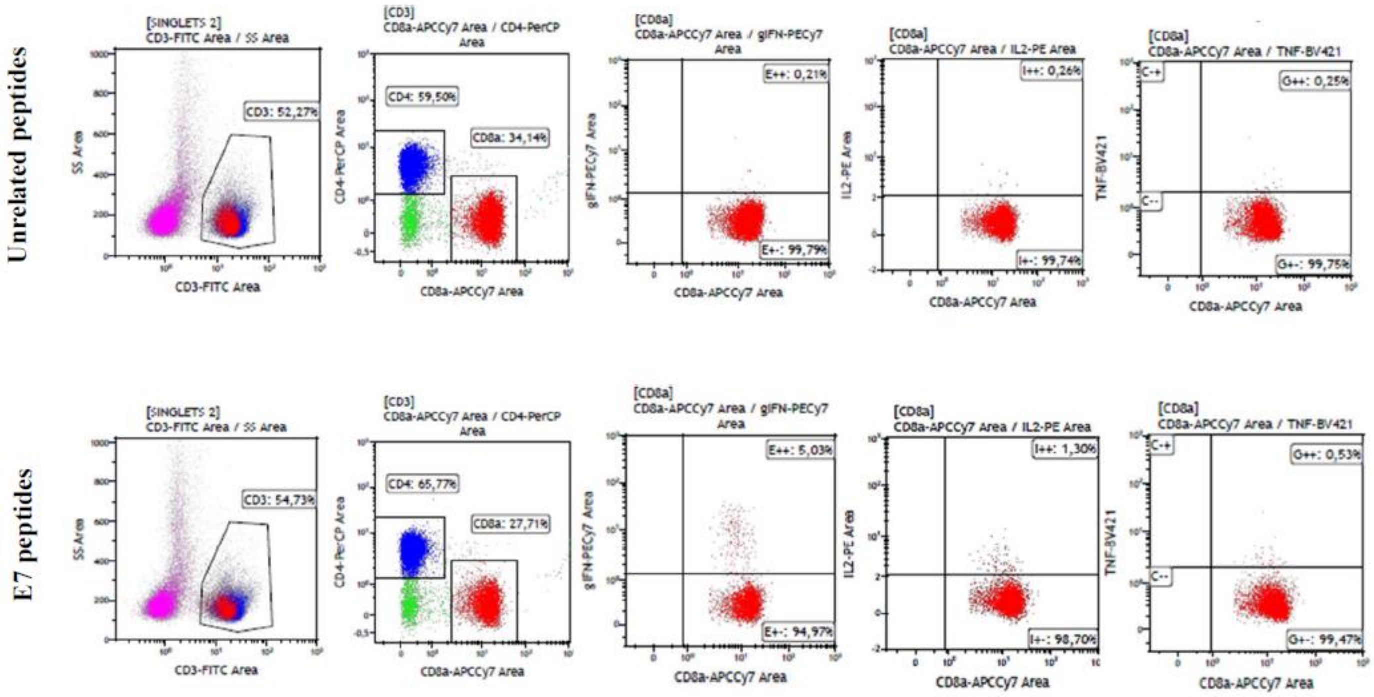

Figure S6. Representative flow cytometer plots from the analysis of splenocytes isolated from Nefmut/E7 injected mice, and treated with either MHC Class I-matched, unrelated nonamers (upper plots) or HPV16 E7-specific nonamers (lower plots).

Splenocytes from Nef^{mut}/E6+Nef^{mut}/E7 injected mice

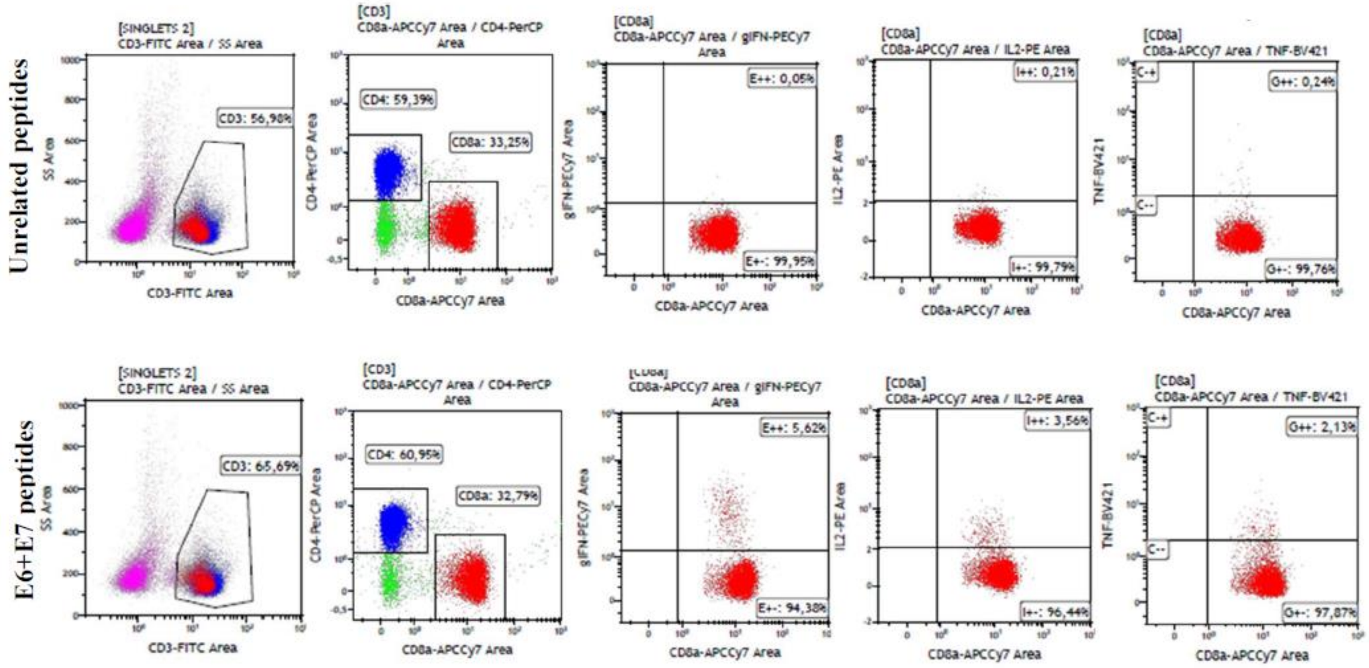

Figure S7. Representative flow cytometer plots from the analysis of splenocytes isolated from mice injected with Nefmut/E6 and Nefmut/E7 vectors, and treated with either MHC Class I-matched, unrelated nonamers (upper plots) or HPV16 E6+E7-specific nonamers (lower plots).

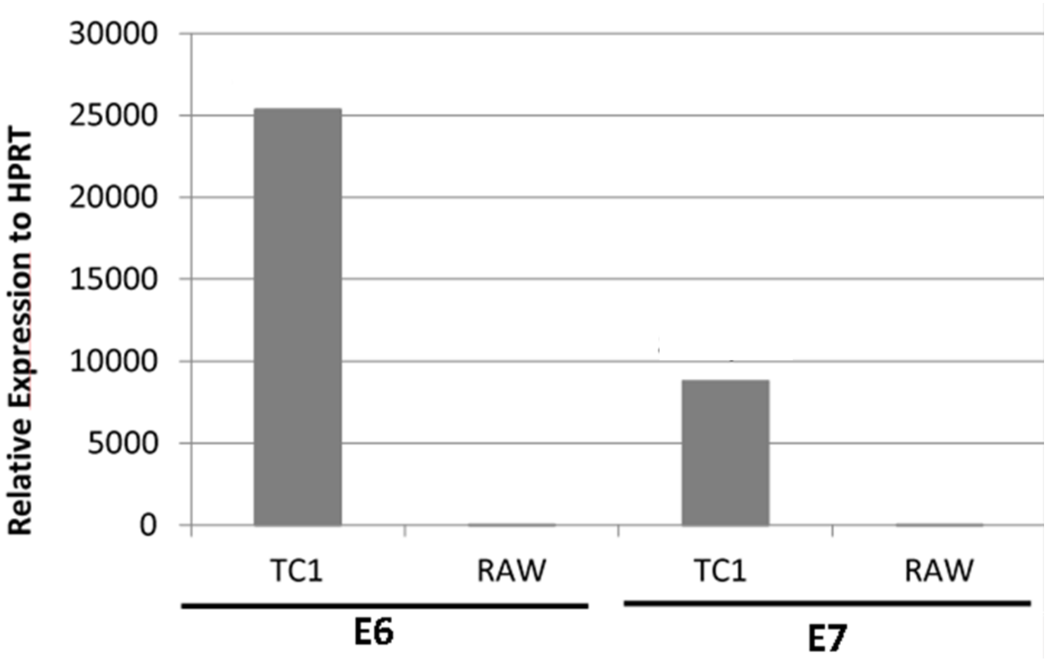

Figure S8. HPV16-E6 and -E7 gene expression in TC-1 cells. HPV16-E6 and -E7 gene expression was quantified by qRT-PCR in TC-1 cells and RAW murine cells as control. Gene expression was normalized by amplifying samples for hypoxanthine guanine phosphoribosyltransferase (HPRT) as house-keeping RNA.

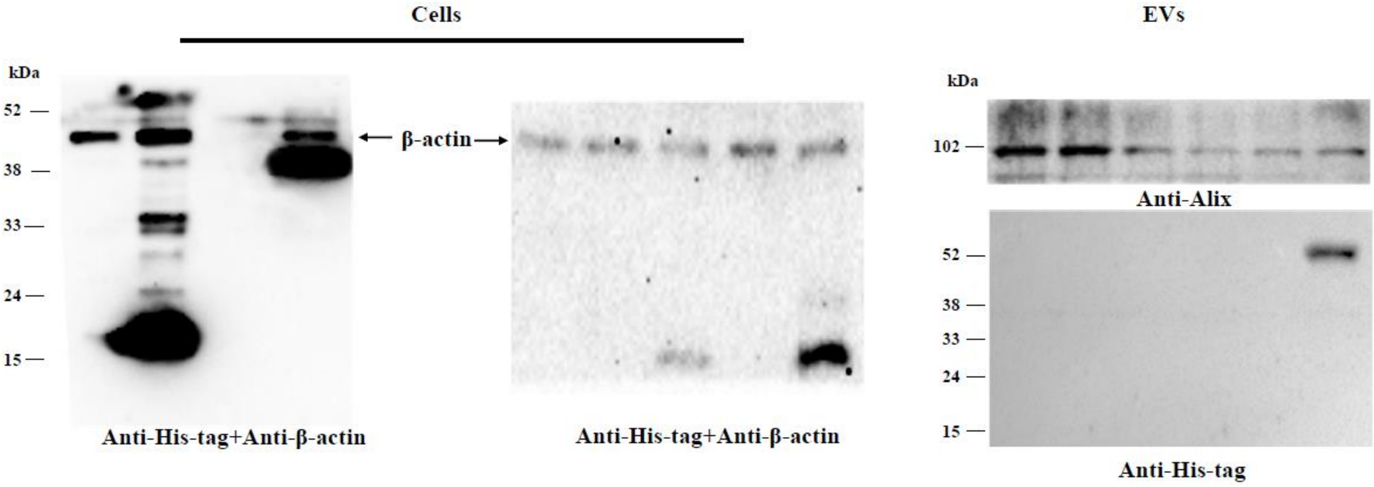

Figure S9. Western blot analysis for the expression of HPV16-E6 and -E7 in HEK293T cells and respective EVs. Raw data.
